# 510. Novel Zwitterionic Hydrogels Reduce Bacterial Burden in a Porcine Infected Burn Model

**DOI:** 10.1093/jbcr/irag033.169

**Published:** 2026-04-06

**Authors:** Amy Wang, Babita Parajuli, Timothy Horseman, Patrick F Walker, David M Burmeister

**Affiliations:** Uniformed Services University, Rockville, MD; Uniformed Services University, Bethesda, MD; US Army Institute of Surgical Research, Schertz, TX; Uniformed Services University, Bethesda, MD; Uniformed Services University, Bethesda, MD

## Abstract

**Introduction:**

Burn injuries impose significant healthcare and logistical burdens in both civilian and military settings. Despite improved survival rates, challenges persist due to the cascade of burn-induced pathophysiologic responses (hypermetabolism, systemic inflammation, and coagulopathy), increasing the risk of infectious complications and delayed healing. These issues are amplified in prolonged field care environments, where definitive clinical care may be delayed beyond 48-72 hours. As such, there is a growing need for adjunctive therapies that mitigate infection and inflammation. Zwitterionic (ZW) hydrogels offer a promising solution by maintaining a moist wound environment, reducing bacterial colonization, and supporting sustained therapeutic delivery. This study evaluates the safety and efficacy of ZW hydrogel loaded with and without polyhexamethylene biguanide (PHMB) in a porcine burn-infection model.

**Methods:**

Ten full thickness 5 × 5 cm contact burns were created on the dorsum of Yorkshire-cross swine (n = 3) with brass probes heated to 100°C, for a total of 30 wounds. A dermal microneedle roller was used to introduce channels in the burns which were inoculated with *Pseudomonas aeruginosa* (2.5 × 10^5^ CFU). Wounds were treated with saline, triple antibiotic ointment (TAO), ZW, or ZW gel + PHMB (n = 7-8/group). Photographs and biopsies were obtained on days 0 and 7 to assess bacterial burden and histology.

**Results:**

On day 7 post-burn, wounds appeared infected due to greenish discoloration of varying degrees. Saline-treated wounds had the highest bacterial burden (9.38 ± 0.40 logCFU/g), which was modestly reduced with TAO to 6.95 ± 0.63 logCFU/g (*p*=.078). Alternatively, ZW and ZW gel + PHMB achieved greater reductions (6.39 ± 0.36 logCFU/g and ~ 5.98 ± 0.65 logCFU/g, respectively; *p<.05 and **p<.01, respectively), indicating superior antimicrobial efficacy. Preliminary histological analysis revealed that negative control (saline-treated) wounds displayed the highest infiltration of white blood cells, particularly within the superficial dermis, which was reduced with both ZW hydrogels (+/- PHMB).

**Conclusions:**

ZW gel formulations (+/- PHMB) significantly reduced bacterial burden in a porcine burn-infection model simulating prolonged field care. While all antimicrobials outperformed saline, ZW-based dressings achieved the greatest reductions, supporting their potential as effective adjuncts in managing infection.

**Applicability of Research to Practice:**

In future large-scale combat operations, disrupted logistical support and limited air superiority may delay casualty evacuation, forcing wounded soldiers to remain in austere environments for extended periods. These conditions pose serious clinical challenges for field medics. Lightweight, multifunctional wound dressings that address infection, inflammation, and delayed healing could significantly improve survivability and outcomes during prolonged field care.

**Funding for the study:**

This work is supported by the Small Business Innovation Research award FA8649-23-P-0744.

